# Thiol-disulfide balance and trace element levels in patients with seasonal allergic rhinitis

**DOI:** 10.4314/ahs.v22i3.34

**Published:** 2022-09

**Authors:** Hasan Basri Savas, Huseyin Gunizi

**Affiliations:** 1 Mardin Artuklu University, Faculty of Medicine, Department of Medical Biochemistry, Mardin, Turkey. Orcid: orcid.org/0000-0001-8759-4507; 2 Alanya Keykubat University, Faculty of Medicine, Department of Ear Nose and Throat, Alanya, Antalya, Turkey. Orcid: orcid.org/0000-0001-8653-0544

**Keywords:** Seasonal allergic rhinitis, native thiol, disulfide, oxidative stress, copper

## Abstract

**Background:**

The prevalence of allergic diseases is gradually increasing worldwide. The most common such allergic disease is allergic rhinitis (AR).

**Objective:**

The present study investigated the possible relationship between seasonal AR and the thiol-disulfide balance and zinc and copper levels in adult individuals.

**Study Design and Methods:**

130 male and female adults were included in the study. The participants' serum thiol-disulfide balance and zinc and copper levels were measured spectrophotometrically using commercial kits. Statistical significance was accepted as p < 0.05 between the groups.

**Results:**

The serum copper (p = 0.001), native thiol (p = 0.006), reduced thiol (p < 0.001), and thiol oxidation reduction ratio (p < 0.001) levels were significantly lower in the seasonal AR group than in the control group.

**Conclusion:**

In AR patients, the low level of copper, which is an important trace element, the deterioration of the thiol-disulfide balance, which represents a unique indicator of the oxidant-antioxidant balance, the increased disulfide level caused by oxidative stress, and the decreased native thiol level can all serve as important biochemical markers.

## Introduction and background

The prevalence of allergic diseases is gradually increasing worldwide. The most common such allergic disease is allergic rhinitis (AR), which has been found to be between 10% and 40% in adults^1^, ^2^. Modern life in the industrialized world is characterized by increased exposure to chemicals and increased exposure to environmental allergens, which contribute significantly to the increase seen in the prevalence of AR. In terms of the mechanism of action behind AR, histamine, interleukin, and prostaglandins all play a role following contact between an allergen substance and the nasal mucosa. More specifically, AR is an inflammatory disease. Indeed, inflammation in the nasal mucosa resulting from the immunoglobulin E (IgE)-dependent hypersensitivity reaction leads to AR. The development of IgE antibodies initiates inflammation by binding to receptors on basophil and mast cells^3^. When the lower and upper respiratory tract is considered whole, allergy can be considered a single disease affecting the respiratory tract. Nasal itching, nasal congestion, and a runny nose represent the classic symptoms of AR. If the symptoms of AR last for more than four weeks in a year and the complaints associated with AR occur for more than four days a week, the condition is known as persistent AR^4^. A significant relationship has been found between seasonal AR in adult individuals and obstructive sleep apnea syndrome, characterized by recurrent upper airway obstruction during sleep. Sleep apnea syndrome can lead to much permanent health problems^5^. AR has significant economic and social impacts due to its high prevalence. It can impair sleep quality. It can also result in a reduced capacity for work. Moreover, its effects may increase seasonally. Steroids and antihistamines can be used to treat AR, although they only provide symptomatic improvement^6^.

The level of reactive oxygen species within the body constantly increases due to the numerous factors that increase oxidative stress. In the human body, any increase in oxidative stress needs to be balanced by increasing the antioxidant capacity. Under normal conditions, reactive oxygen species are deactivated by the components of the antioxidant system, which results in health being maintained. Yet, reactive oxygen species begin to accumulate if there is an excessive increase in oxidative stress, or a marked decrease in the antioxidant capacity, and the balance is broken. The accumulation of the products of oxidative stress can cause permanent damage to the cell membrane, nuclear membrane, organelles, and genetic material. Copious serious diseases occur because of the cell, tissue, and organ damage. Many parameters can be measured to evaluate the degree of oxidative stress and antioxidant capacity. The balance between native thiol and disulfide is considered a good indicator of the oxidant-antioxidant balance. An unbalanced increase in the reactive oxygen species increases the conversion of native thiol into disulfide^7^–^16^. Although it is associated with asthma, the thioldisulfide balance has not yet been adequately studied concerning seasonal AR, despite the disease being quite common in adults^17^. In addition, the levels of zinc and copper, which are both important trace elements and cofactors of many antioxidant enzymes, are likely to affect the thiol-disulfide balance in patients with seasonal AR. The present study investigated the possible relationship between seasonal AR and the thiol-disulfide balance and zinc and copper levels in adult individuals.

## Methods

### Study Design

A total of 130 male and female adults were included in the present study. The experimental group was formed by obtaining voluntary informed consent to participate from 65 adult patients who had been diagnosed with seasonal AR. The control group was formed by obtaining voluntary informed consent to participate from 65 healthy adults who did not suffer from any allergic diseases. Sera leftover from routine tests were collected and stored at-80°C. The sera were dissolved by bringing them up to room temperature during the study time. The sera were mixed using a vortex device. Simultaneously, the thiol-disulfide balance and zinc and copper levels were measured spectrophotometrically in all the samples using commercial kits. The remaining descriptive and clinical information were retrieved from the patients' files.

### Ethical Issues

All the procedures applied in the present study were conducted in accordance with the ethical requirements of the Declaration of Helsinki. In addition, permission to conduct the research was granted by the ALKU Clinical Research Ethics Committee (date: 22.04.2020, number: 18-4).

### Thiol-Disulfide Balance

The serum thiol-disulfide balance was examined spectrophotometrically. The measurement process was performed according to a new method described in the literature16. If the method for thiol measurement is briefly described, the disulfide bonds are first reduced to form free functional thiol groups. Meanwhile, the reducing sodium borohydride not used in the reaction was removed with formaldehyde. Then, all thiol groups containing natural thiol groups were determined as a result of the reaction with DTNB (5, 5-dithiobis-2-nitrobenzoic acid).^16^ Commercial kits were used (Rel Assay Diagnostics, Gaziantep, TURKEY) for all the measurements. The natural (native) thiol (reduced thiol) and total thiol (reduced thiol and oxidized disulfide bonds) levels were also measured. The amount of dynamic disulfide is equivalent to the difference between half the total thiol level and the natural thiol level^16^. The following rates were then calculated. Reduced Thiol = (Native Thiol / Total Thiol) * 100. Oxidized Thiol = (Disulfide / Total Thiol) * 100. Thiol Oxidation Reduction Ratio = (Native Thiol / Disulfide) * 100. Units: Disulfide levels (µmol / l). Total thiol (µmol / l) / alb (g / l). Native thiol (µmol / L) / alb (g / l). Disulfide (µmol / l) / alb (g / l)^16^.

### Zinc Levels

Serum zinc levels were measured with the colorimetric method defined in the literature with the Rel Assay Diagnostics (Gaziantep, TURKEY) brand commercial kit. Briefly, the color of 5-Br-PAPS turned from red-orange to light pink caused by the zinc present in the specimens under alkaline conditions. The absorbance level proportionally alters with the overall zinc content in the samples when at 548 nm. The experimental calibration was carried out by dissolution of zinc sulfate in deionized water. The units are given as µg/dl 18.

### Copper Levels

Serum copper levels were measured with the colorimetric method defined in the literature with a commercial kit from Rel Assay Diagnostics (Gaziantep, TURKEY). Briefly, the color of DiBr-PAESA turned from copper found red-orange to violet by copper present in specimens under acidic conditions. The absorbance level proportionally alters with the overall copper content in the samples when at 572 nm. The experimental calibration was carried out by dissolution of copper sulfate in deionized water. The units are given as µg/dl^19^.

### Statistical analysis

Thiol balance, disulfide, and trace element levels were statistically compared between the control and seasonal allergic rhinitis groups. All necessary statistical analyse were performed using the IBM Statistical Package Social Sciences (SPSS) 21.0 (IBM, New York, USA) computer program. Categorical variables were given as numbers and percentages. Continuous variables were given as mean and standard deviation. ANOVA test was applied. The statistical significance level was accepted as 0.05 for all tests.

## Results

The age distribution of the groups in mean ± standard deviation was control (n = 65): 35.4 ± 13.76 and seasonal allergic rhinitis (n = 65): 35.69 ± 13.68. The seasonal allergic rhinitis group consisted of a total of 65 patients, 39 women, and 26 men. Control group; It consisted of a total of 65 patients, 38 women, 27 men.

Serum copper (p=0.001), native thiol (p=0.006), reduced thiol (p<0.001), thiol oxidation-reduction ratio (p<0.001) levels were significantly lower in the seasonal allergic rhinitis group compared to the control group. Serum disulfide (p<0.001) and oxidized thiol (p<0.001) levels were significantly higher in the seasonal allergic rhinitis group compared to the control group. Descriptive statistics and ANOVA test results are shown in table 1.

**Table 1:** Descriptive and Compared Statistical Analyses

Parameters / Units		N	Mean	Std. Deviation	Std. Error	95% Confidence Interval for Mean		Anova
						Lower Bound	Upper Bound	*p*
**Age**	Control	65	35.40	13.76	1.71	31.99	38.81	.904
	Seasonal Allergic Rhinitis	65	35.69	13.68	1.70	32.30	39.08	
	Total	130	35.55	13.67	1.20	33.17	37.92	
**Zn**	Control	65	84.86	8.07	1.00	82.86	86.86	.246
	Seasonal Allergic Rhinitis	65	87.08	13.07	1.62	83.85	90.32	
	Total	130	85.97	10.88	0.95	84.09	87.86	
**Cu**	Control	65	134.08	43.74	5.43	123.24	144.92	**.001** *
	Seasonal Allergic Rhinitis	65	109.74	37.22	4.62	100.51	118.96	
	Total	130	121.91	42.26	3.71	114.57	129.24	
**Total** **Thiol**	Control	65	612.11	86.61	10.74	590.65	633.57	.522
	Seasonal Allergic Rhinitis	65	601.94	93.88	11.64	578.68	625.21	
	Total	130	607.03	90.11	7.90	591.39	622.66	
**Native** **Thiol**	Control	65	481.88	85.16	10.56	460.77	502.98	**.006** *
	Seasonal Allergic Rhinitis	65	437.44	94.90	11.77	413.92	460.95	
	Total	130	459.66	92.54	8.12	443.60	475.72	
**Disulfide**	Control	65	65.12	21.55	2.67	59.78	70.45	**.000** *
	Seasonal Allergic Rhinitis	65	82.25	20.18	2.50	77.25	87.25 *	
	Total	130	73.68	22.50	1.97	69.78	77.59	
**Reduced Thiol**	Control	65	78.58	6.87	0.85	76.87	80.28	**.000** *
	Seasonal Allergic Rhinitis	65	72.16	7.53	0.93	70.30	74.03	
	Total	130	75.37	7.87	0.69	74.00	76.74	
**Oxidized Thiol**	Control	65	10.70	3.44	0.43	9.84	11.55	**.000** *
	Seasonal Allergic Rhinitis	65	13.92	3.76	0.47	12.99	14.85	
	Total	130	12.31	3.94	0.35	11.63	12.99	
**Thiol Oxid. Red. Ratio**	Control	65	824.58	307.22	38.11	748.45	900.70	**.000** *
	Seasonal Allergic Rhinitis	65	575.36	232.70	28.86	517.70	633.02	
	Total	130	699.97	298.90	26.22	648.10	751.83	

* p<0.05. Statistically significant difference.

**Figure 1:**
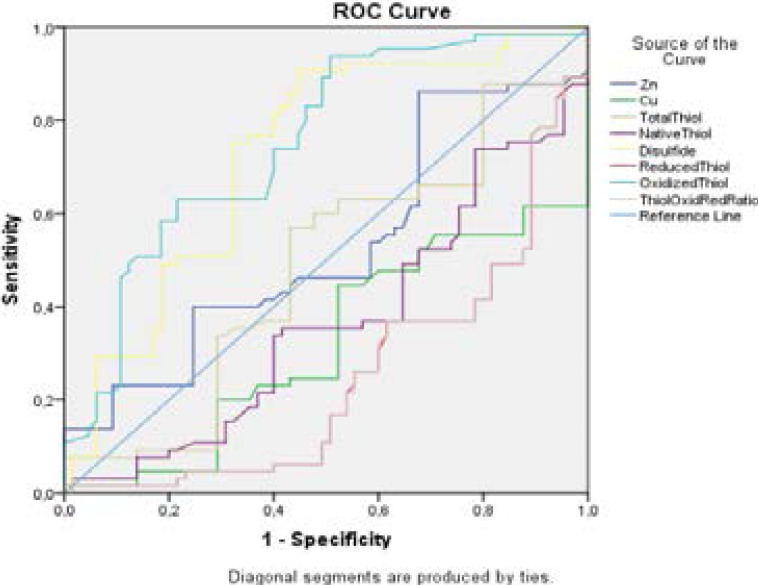
ROC Curve for Laboratory Parameters

In addition, routine biochemistry parameters belonging to the seasonal allergic rhinitis group were taken from their files. Routine biochemical parameters were analyzed according to reference ranges. Ig E levels were above the reference range. The results of routine biochemical parameters are given in table 2. ROC Curve for laboratory parameters is shown in figure 1. The area under the ROC Curve for laboratory parameters is shown in table 3.

**Table 2:** Routine Biochemical Parameters of Seasonal Allergic Rhinitis Group

	Unit	Mean	Std. Deviation	Reference Range
**Leukocyte**	cells per microliter of blood	7,66	2,22	3.7–10.1
**Lymphocyte**	cells per microliter of blood	2,37	0,83	1.09–2.99
**Neutrophil**	cells per microliter of blood	4,49	1,54	1.63–6.96
**Neutrophil/ Lymphocyte**	Ratio	2,03	0,82	Ratio
**Eosinophil**	cells per microliter of blood	0,19	0,15	0.03–0.44
**Basophil**	cells per microliter of blood	0,05	0,03	0–0.08
**Hemoglobin**	g/dL	13,44	2,00	12.9–15.9
**Vitamin B12**	ng/L	371,86	224,45	197–771
**Ferritin**	microg/L	49,03	45,38	30–400
**Free t3**	pmol/L	2,90	0,34	3.1–6.8
**Free t4**	mol/L	1,51	0,99	12–22
**TSH**	mU/L	2,17	1,39	0.27–4.2
**Ig E**	U/L	177,79	180,20	**1–100** *

* Refers to the higher value compared to the reference range.

**Table 3:** Area Under the ROC Curve for Laboratory Parameters

Area Under the Curve					
Test Result Variable(s)	Area	Std. Error^a^	Asymptotic Sig.^b^	Asymptotic 95% Confidence Interval	
				Lower Bound	Upper Bound
Zn	.528	.052	.580	.427	.629
Cu	.319	.047	.000	.227	.411
Total Thiol	.482	.052	.727	.381	.583
Native Thiol	.367	.049	.009	.272	.462
Disulfide	.728	.045	.000	.639	.817
Reduced Thiol	.249	.043	.000	.165	.333
Oxidized Thiol	.752	.043	.000	.668	.836
Thiol Oxidation Reduction Ratio	.249	.043	.000	.165	.332

The test result variable(s): Zn, Cu,

Total Thiol, Native Thiol, Disulfide, Reduced Thiol, Oxidized Thiol, Thiol Oxidation Reduction Ratio has at least one tie between the positive actual state group and the negative actual state group. Statistics may be biased.

a Under the nonparametric assumption

b Null hypothesis: true area = 0.5

ROC Curve for laboratory parameters is shown in figure 1. The area under the ROC Curve for laboratory parameters is shown in table 3.

The area under the receiver operating curve (ROC) curve (AUC-ROC)is used as a method to show the accuracy of diagnostic tests. The larger the area under the curve, the better the test at distinguishing patients. The ideal value for AUC is 1. The line drawn at an angle of 45 degrees from the zero point is considered the reference line.

## Discussion

As a result of our research, a significant decrease in native thiol (p=0.006) and copper (p=0.001) levels and a significant increase in disulfide (p<0.001) levels in seasonal adult allergic rhinitis patients indicate an increase in oxidative stress.

Aerobic organisms constantly use oxygen for the continuation of life. During oxygen consumption, the most important free radicals in the body are formed. Reactive oxygen species are released in the form of superoxide, hydrogen peroxide, and hydroxyl radical. These free radicals are highly reactive and can cause many cellular damages, including the genetic material. Various effects occur on biomolecules in the form of nonenzymatic lipid peroxidation, structural and functional changes in amino acids and proteins, oxidative DNA damage and mutations, the formation of oxidized monosaccharides, and oxoaldehydes. To prevent this oxidative stress increase process from turning into disease and damage, the antioxidant system components are very important and continuously work to balance and neutralize oxidative stress. The most important enzymatic components of the antioxidant system are superoxide dismutase (SOD), glutathione peroxidase (GPx), glutathione S-transferase (GST), catalase (CAT), paraoxonase (PON), and mitochondrial cytochrome oxidase system. In addition, there are vitamins such as alpha-tocopherol, ascorbic acid, beta carotene, and folic acid, and various nonenzymatic antioxidants such as melatonin, ferritin, albumin, glutathione, myoglobin, and hemoglobin. If the activities of all these antioxidants are not enough to neutralize oxidative stress, many serious and deadly diseases can result^7^–^16^. Thiol-disulfide balance is one of the unique and new biochemical markers that can show the oxidant-antioxidant balance^16^. Allergic rhinitis is an important disease that can reach 40% in adults and negatively affect the quality of life^3^. However, there is not enough comprehensive clinical research in the literature on the relationship between thiol-disulfide balance and adult allergic rhinitis. For this reason, the disulfide increase and native thiol decrease revealed in allergic rhinitis patients in our research are very important. There are various studies in the literature investigating the role of oxidative stress in the etiology of ear, nose, and throat (ENT) diseases. It has been reported to play a role in the etiology of hearing loss, rhinosinusitis, otitis media, chronic tonsillitis, and laryngeal cancer^20^–^23^. They suggested that oxidative stress increases, and benign paroxysmal positional vertigo (BPVV) develop in patients with vitamin D deficiency^24^.

Anti-inflammatory agents are given to end the developing inflammatory process also function on this basis. Sino-nasal Outcome Test (SNOT)-22 score is a subjective test used to evaluate allergic rhinitis. In a study, they found a lower SNOT-22 score and serum oxidative stress level after AR attack compared to pre-attack^25^. Ulusoy et al. found that the disulfide level was significantly higher in patients during the attack. In their study on plasma thiol level in 32 adult patients with AR they found that the native thiol level was significantly higher in the asymptomatic period^23^. This study supports our research results. In addition, our results are statistically more valuable since the number of participants in the patient and control groups in our study is higher.

Serum Zn and Cu values can give information about antioxidant capacity. For example, Liu et al., in their studies on ARs, found serum Zn levels significantly lower in the patient group than in the control group^26^. In another study, they found that the Znspan level was statistically significantly lower in the patient group and the Cu level was higher in the patient group^27^.

In our research results, Ig E values above the reference range in the AR group confirm the diagnosis of allergic rhinitis. Therefore, evidence-based diagnosis of AR should be considered in terms of the value of the study when interpreting the change in thiol-disulfide balance1–3. Other routine biochemical laboratory parameters are within reference ranges. For this reason, they can be considered normal. The results are shown in table 2.

Studies investigating thiol-disulfide balance and trace element levels in allergic rhinitis patients are scarce in the literature. However, in the current literature, it has been shown that thiol-disulfide balance may be a clinical indicator in many diseases with uncertain etiology^28^, ^29^. Our study showed that blood copper levels and thiol thiol-disulfide balance might be a clinical laboratory indicator in allergic rhinitis patients. New clinical studies should confirm thiol-disulfide balance and trace element changes in AR patients.

## Conclusion, recommendations, and future directions

In AR patients, the low level of copper, which is an important trace element, the deterioration of the thiol-disulfide balance, which represents a unique indicator of the oxidant-antioxidant balance, the increased disulfide level caused by oxidative stress, and the decreased native thiol level can all serve as important biochemical markers. Thus, copper, disulfide, and thiol levels can be practical biochemical indicators in diagnosis, treatment, and follow-up in allergic rhinitis patients. In addition, nutritional and lifestyle changes in allergic rhinitis patients may provide significant clinical benefits for increasing antioxidant capacity.
